# Prognostic Factors of the Efficacy of High-dose Corticosteroid Therapy in Hemolysis, Elevated Liver Enzymes, and Low Platelet Count Syndrome During Pregnancy: A Meta-analysis

**DOI:** 10.1097/MD.0000000000003203

**Published:** 2016-04-01

**Authors:** Li Yang, Chenchen Ren, Minhong Mao, Shihong Cui

**Affiliations:** From the Department of Gynecology & Obstetrics (LY, CR, SC), The Third Affiliated Hospital of Zhengzhou University, Zhengzhou, Henan; and West Zone (MM), Beijing Chaoyang Hospital, Capital Medical University, Beijing, China.

## Abstract

The aim of this study was to identify the factors which can affect the efficacy of corticosteroid (CORT) therapy in the management of hemolysis, elevated liver enzymes, and low platelet count (HELLP) syndrome.

Research articles reporting the efficacy of CORT therapy to HELLP syndrome patients were searched in several electronic databases including EMBASE, Google Scholar, Ovid SP, PubMed, and Web of Science. Study selection was based on predefined eligibility criteria. Efficacy was defined by the changes from baseline in HELLP syndrome indicators after CORT therapy. Meta-analyses were carried out with Stata software.

Data of 778 CORT-treated HELLP syndrome patients recruited in 22 studies were used in the analyses. Corticosteroid treatment to HELLP syndrome patients was associated with significant changes from baseline in platelet count; serum levels of aspartate aminotransaminase, alanine transaminase, and lactic dehydrogenase (LDH); mean blood pressure; and urinary output. Lower baseline platelet count predicted higher change in platelet count after CORT therapy. Lower baseline platelet count and lower baseline urinary output predicted greater changes in LDH levels after CORT therapy. There was also an inverse relationship between the change from baseline in LDH levels and intensive care duration. Higher CORT doses were associated with greater declines in the aspartate aminotransaminase, alanine transaminase, and LDH levels. Incidence of cesarean delivery was inversely associated with the gestation age. The percentage of nulliparous women had a positive association with the intensive care stay duration.

High-dose CORT therapy to HELLP syndrome patients provides benefits in improving disease markers and reducing intensive care duration, especially in cases such as mothers with much lower baseline platelet count and LDH levels.

## INTRODUCTION

Hemolysis, elevated liver enzymes, and low platelet count (HELLP) syndrome is a potentially lethal consequence of gestational hypertension. Pathological dynamics of the syndrome, that is, microangiopathic hemolytic anemia, liver dysfunction, and thrombocytopenia are not easier to understand at the earliest and therefore diagnosis is often late.^1–4^

Three main options for the management of HELLP syndrome are as follows: immediate delivery at 34 weeks’ gestation or later; delivery within 48 hours after evaluation and stabilization of the maternal clinical condition and corticosteroid (CORT) treatment during 27 to 34 weeks of gestation; and conservative management for more than 48 to 72 hours in pregnant women before 27 weeks’ gestation.^5^

Use of CORT in the management of HELLP syndrome is frequent. Data generated in the past 2 decades suggest that the CORT treatment to HELLP patients is associated with improvements in biological markers including platelet count, serum aspartate aminotransaminase (AST), alanine transaminase (ALT), and lactic dehydrogenase (LDH) levels, without affecting overall morbidity and mortality.^6,7^ It is important to know which factors can affect the efficacy of CORT in improving HELLP syndrome markers to identify subgroups of patients that can be benefitted with CORT treatment. We therefore carried out a systematic review and metaregression analysis to identify the factors affecting the efficacy of CORT therapy by using data generated in the studies, which evaluated the efficacy of CORT therapy in HELLP patients.

## METHOD

Preferred Reporting Items for Systematic Reviews and Meta-Analyses (PRISMA) guidelines are followed for carrying out the present study.^8^ Eligibility criteria for the selection of studies are presented in Table 1. Ethical approval is not required for a meta-analysis.

**TABLE 1:**
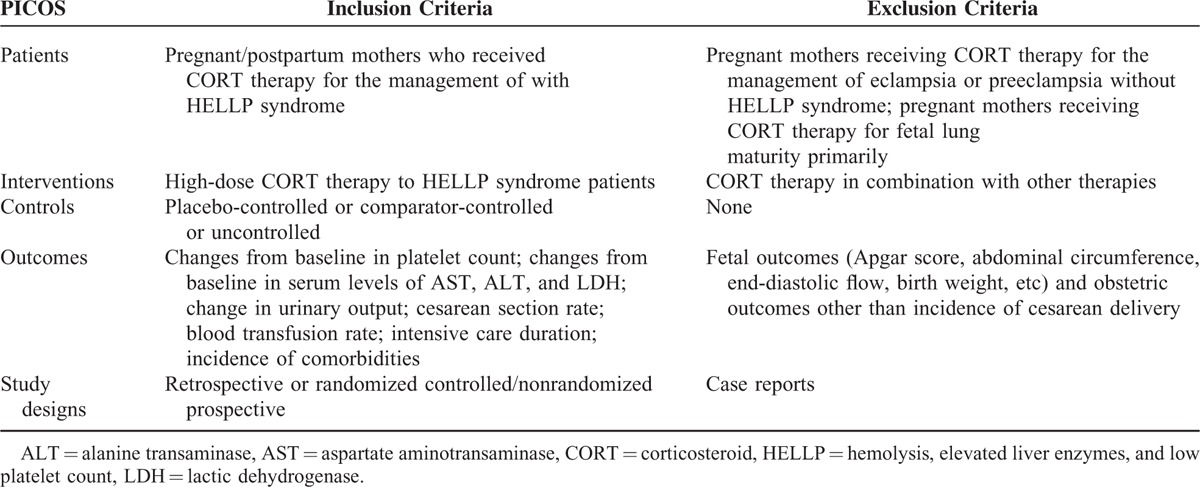
Study Eligibility Criteria

### Literature Search

Several electronic databases including EMBASE, Google Scholar, Ovid SP, PubMed/Medline, and ASI Web of Science were searched for original research articles published before July 2015. The Medical Subject Headings and keywords specific to this research, including hemolysis, elevated liver enzymes, and low platelet count (HELLP) syndrome, corticosteroid therapy, dexamethasone, betamethasone, prednisolone, peripartum, antepartum, postpartum, lactic dehydrogenase (LDH), aspartate aminotransaminase (AST), and alanine transaminase (ALT), were used in logical combinations and phrases. Search strategy is described in the supplementary information file. Additional methods of search included the scrolling of cross-references of important research articles and exploration of the software corroborations.

### Data Extraction, Synthesis, and Analyses

Information regarding the endpoints and outcomes, dosage and mode of CORT administration, and clinical, obstetric, perinatal, and demographic characteristics were obtained from identified research articles and organized in Microsoft Excel datasheets. For estimation of the efficacy of CORT therapy, changes from baseline in disease indicators were extracted from respective research reports or calculated from raw data, if not provided. For studies which did not provide standard deviation values of the changes from baseline, these were imputed by using formulae that yield calculate the nearest approximate estimates.^9^

Metaregression analyses were carried out with Stata software (version 12; College Station, TX). Restricted maximum likelihood method was used for the metaregression analyses. Between-study variance was tested with tau^2^ and the percentage of between-study heterogeneity was assessed with I^2^ index. For the identification of the predictors and prognostic factors of the efficacy of CORT therapy, the analyses were carried out systematically by testing each of the dependent variables (changes from baseline in the platelet count, AST, ALT, and LDH, change in urinary output, intensive care duration, and the incidence of cesarean deliveries) against several independent variables including the number of patients in a study, CORT total dose, mother's age, nulliparity, gestation week at the time of CORT treatment, baseline platelet count, baseline mean blood pressure, baseline urinary output, baseline AST levels, baseline ALT levels, and baseline LDH levels. A *P* value of less than 0.1 was considered to show a significant relationship.

For the assessment of publication bias, Begg funnel plot and Egger precision plot were studied, and trim and filled method was applied to estimate missing number of studies.

## RESULTS

**FIGURE 1:**
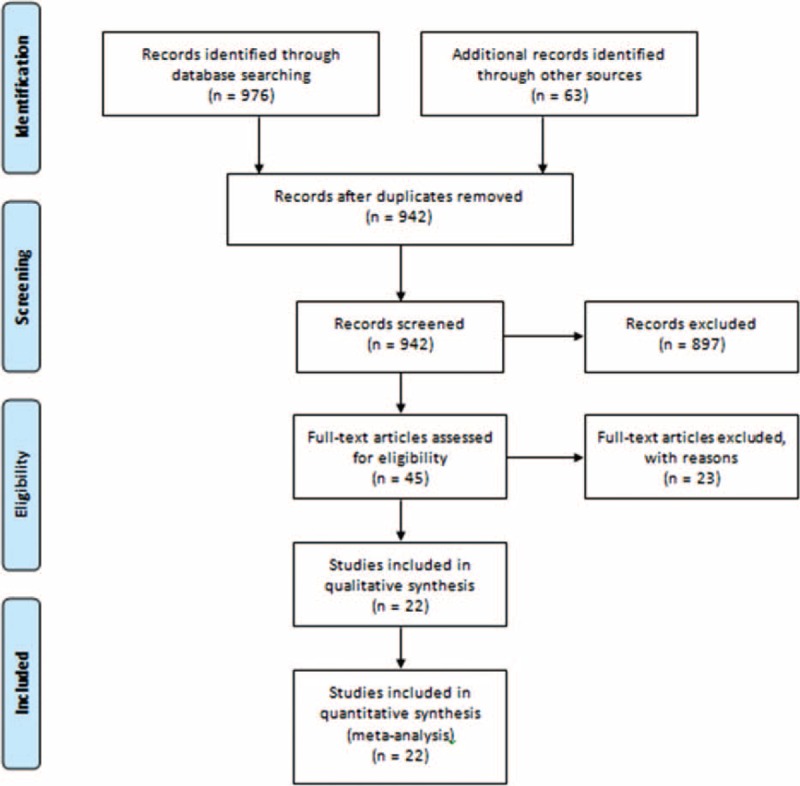
A flowchart of study screening and selection process.

**FIGURE 2:**
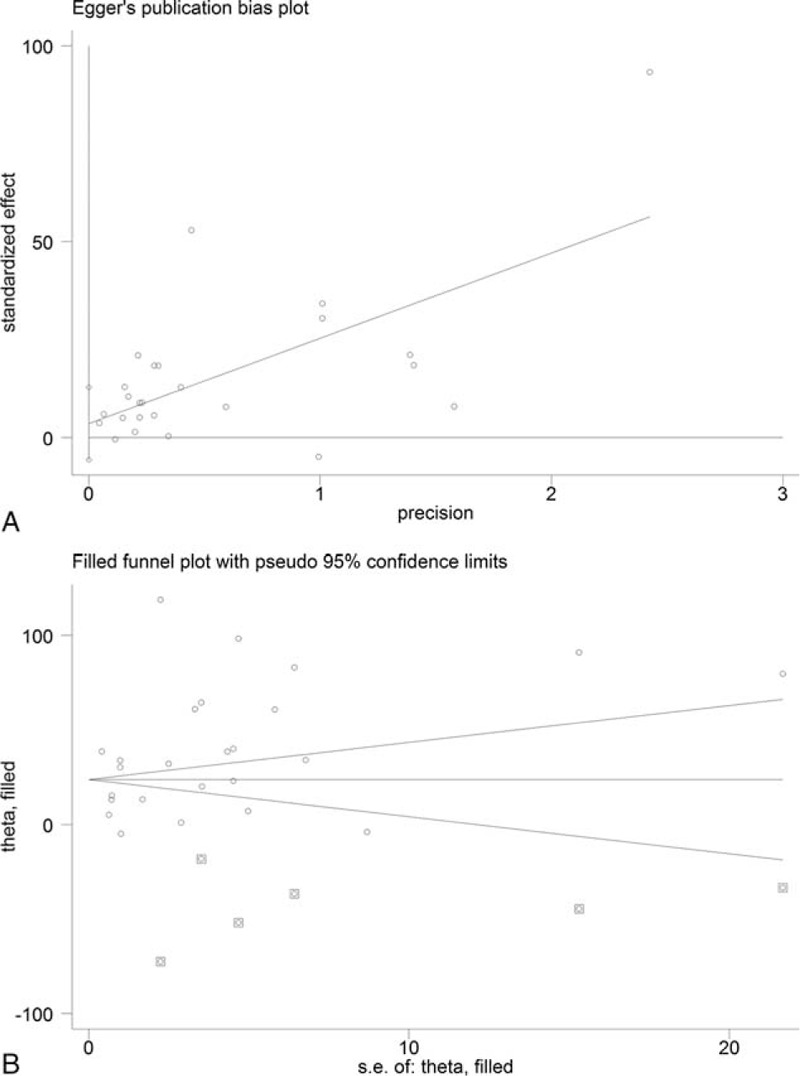
Publication bias assessment charts corresponding to the meta-analysis of the change from baseline in platelet count after CORT therapy to HELLP syndrome patients: A, Egger plot, and B, Begg funnel plot with trim and fill procedure. CORT = corticosteroid, HELLP = hemolysis, elevated liver enzymes, and low platelet count.

Twenty-two studies,^10–31^ which overall recruited 778 CORT-treated patients, were used for the analyses (Figure 1). Of the included studies, 10 were randomized controlled trials (RCTs), 1 nonrandomized prospective study, and 11 were retrospective analyses. Significant publication bias was observed with the Egger and Begg methods of assessment (Figure 2A and B). The CORT was administered during antepartum period in 9 studies, during postpartum period in 8 studies, and during peripartum period in 5 studies.

Age of the patients as mean ± SD (range) was 26.12 ± 2.64 (23.2 ± 6 to 33.5 ± 4) years. Gestation age at the time of randomization or CORT treatment was 31.6 ± 2.22 (range 27 ± 1–35.1 ± 2.9) weeks. Of these patients, 41.6 ± 31.4% women were nulliparous. Total dose administered to these patients ranged from 30 to 150 mg, with an average of 60.8 ± 45.5 mg (12 studies data). Incidence of cesarean deliveries in CORT-treated HELLP syndrome patients was 68.9 ± 19.4% (range 33.3%–100%).

**FIGURE 3:**
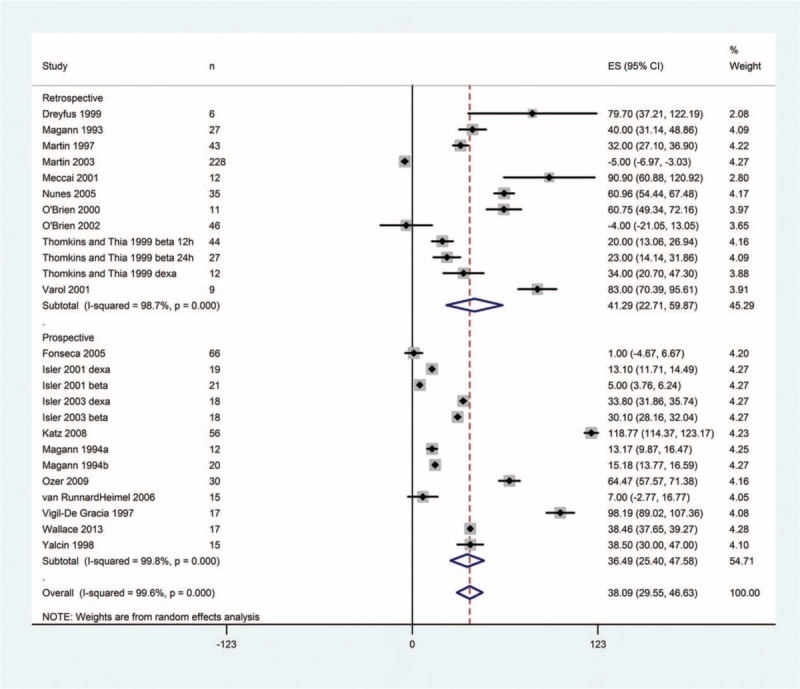
Forest graph showing the meta-analysis of the change from baseline in platelet count in CORT-treated HELLP syndrome patients. CORT = corticosteroid, HELLP = hemolysis, elevated liver enzymes, and low platelet count.

Important findings of the meta-analysis are presented in Tables 2 and 3, and the results of all systematic metaregression analyses are given in supplementary information file (Tables S1–S7). Corticosteroid treatment to HELLP syndrome patients was associated with significant improvements in the changes from baseline in platelet count, serum levels of AST, ALT, and LDH, mean blood pressure, and urinary output (Figure 3, Table 2, and Figures S1–S5).

**TABLE 2:**
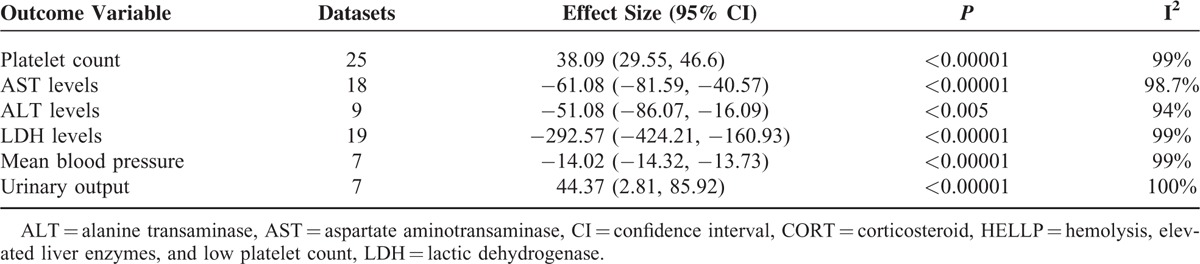
Effect Sizes (Changes From Baseline) in the Markers of HELLP Syndrome in CORT-treated Patients

**TABLE 3:**
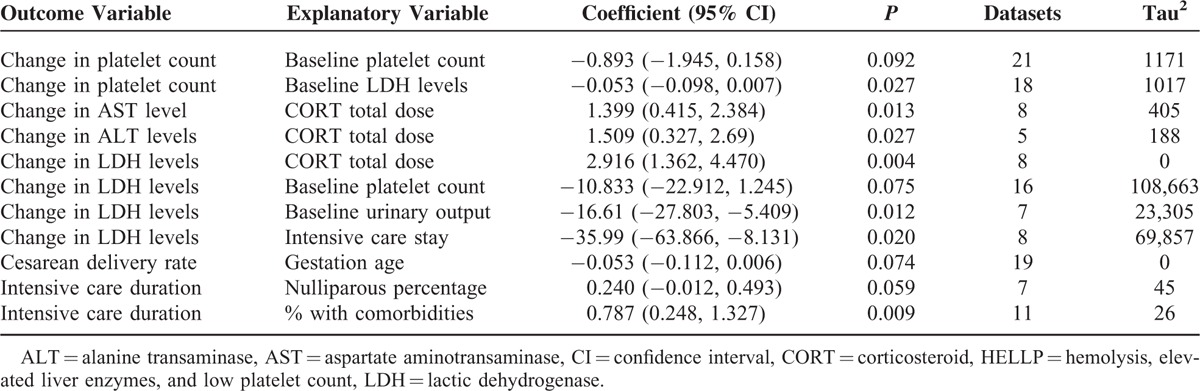
Important Findings of the Metaregression Analyses

**FIGURE 4:**
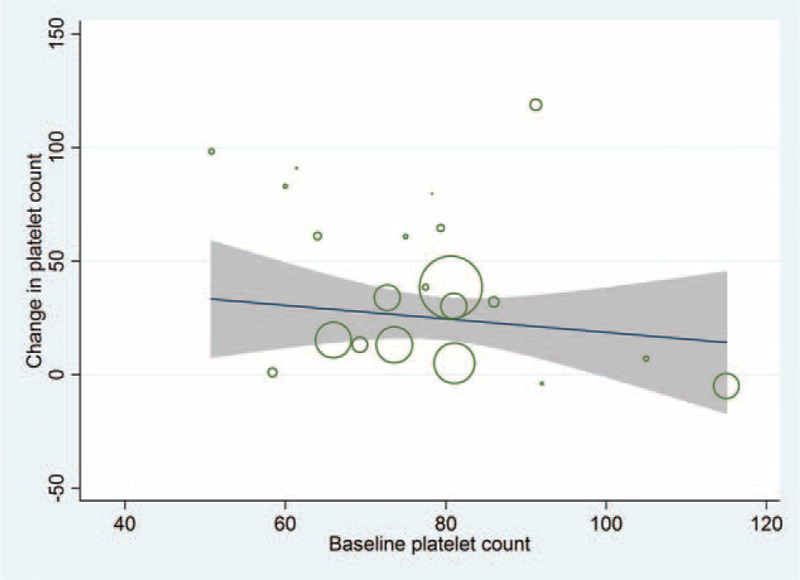
Scatter plot showing the relationship between the change from baseline in platelet count after CORT therapy, and (A) baseline platelet count and (B) baseline LDH levels. CORT = corticosteroid, LDH = lactic dehydrogenase.

Change in platelet count from baseline had a significant inverse relationship with baseline platelet count (Figure 4, Table 3). Thus, lower baseline platelet count and lower baseline LDH levels were predicting higher level change in platelet count from the CORT therapy. However, there was also an inverse relationship between baseline LDH levels and the change in platelet count after CORT therapy. Thus, lower baseline LDH levels were predicting higher change in platelet count.

Lower baseline platelet count and lower baseline urinary output also predicted higher changes in LDH levels after CORT therapy as a significant inverse relationship was observed between the changes from baseline in LDH levels and the baseline platelet count or urinary output (Table 3). There was also an inverse relationship between the change from baseline in LDH levels and intensive care duration.

Total CORT dose had significantly positive association with the changes from baseline in AST, ALT, and LDH levels. Thus, higher CORT doses were associated with more declines in these enzyme levels (Table 3).

**FIGURE 5:**
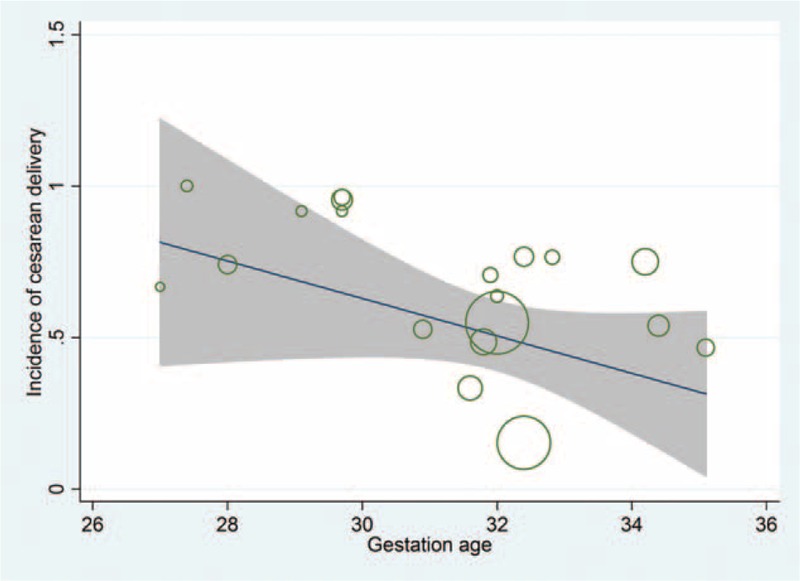
Plots showing the relationship between the incidence of cesarean deliveries and gestation age in the CORT-treated HELLP syndrome patients. CORT = corticosteroid, HELLP = hemolysis, elevated liver enzymes, and low platelet count.

Incidence of cesarean delivery was inversely associated with the gestation age (Figure 5). The intensive care stay duration was positively associated with the percentage of nulliparous women and the percentage of HELLP syndrome patients with comorbidities (Table 3). None of the other independent variables were significantly associated with the incidence of cesarean delivery (Tables S1–S7).

## DISCUSSION

This pooled analysis, followed by the metaregression analyses, has found that the CORT treatment to HELLP syndrome patients significantly improves platelet count; AST, ALT, and LDH levels; and urinary output. Changes in platelet count and LDH levels after CORT therapy were more robust in patients with lower baseline platelet count, and these outcomes were also associated with lesser intensive care duration. However, intensive care duration was also positively associated with nulliparity. Cesarean delivery rate was negatively associated with gestation age.

Class 1 HELLP syndrome is associated with relatively higher maternal mortality and poor prognosis in delayed diagnosis cases.^32^ A statistically significant negative association between the change from baseline in platelet count and the baseline platelet count, and also baseline LDH levels observed in the present study suggests that CORT treatment can be more efficacious in patients with lower platelet count and thus CORT therapy to class 1 patients can be more beneficial. In a RCT also, a faster platelet recovery was found in the class 1 subgroup of HELLP patients after CORT therapy.^11^ After analyzing HELLP class 1 patients’ data retrospectively, Vigil-De Gracia^29^ also suggested high-dose CORT therapy for class 1 HELLP syndrome patients.

In the present analysis, we have found that the incidence of cesarean deliveries was inversely associated with the gestation week, and intensive care duration was positively associated with the percentage of nulliparous women in the CORT-treated HELLP syndrome patients. Neither any of the baseline biological marker nor change in the biological markers after CORT therapy was significantly associated with cesarean delivery rate. Recently, Katz et al,^33^ after analyzing 500 women with severe preeclampsia, have observed that whereas uncontrolled hypertension was a risk factor for cesarean section, there was no association between cesarean section and HELLP syndrome. Haddad et al,^34^ in a retrospective analysis of 183 HELLP syndrome cases, have found that except for the need for blood products transfusion, onset of HELLP syndrome at or less than 28 weeks’ gestation was not associated with an increased risk of adverse maternal or neonatal outcomes when they compared these cases with severe preeclampsia patients without the HELLP syndrome at a similar gestational age.

Haddad et al,^35^ in the same 183 HELLP syndrome cases, found that women with nadir platelet counts <50,000 cells/μL were at increased risk for adverse maternal outcomes in comparison with those with nadir platelet counts between 50,000 and 100,000 cells/μL. However, their logistic regression analyses could not identify significant associations between the nadir values of platelet count of <50,000/μL, serum AST level of >150 U/L, or serum LDH level of >1400 IU/L and maternal adverse outcome. There was also no significant association between laboratory markers and the adverse outcomes in a study of 44 HELLP syndrome patients.^36^ The present study also could not find any significant relationship between baseline platelet count, AST, ALT, or LDH, and the incidence of comorbidities in CORT-treated HELLP syndrome patients. Intensive care duration was negatively associated with gestation week and positively associated with nulliparity and percent patients having comorbidities in HELLP patients, but not with the severity of biological markers per se.

Some of the limitations of the present study may have compromised the overall outcomes to some extent. Firstly, studies with varying designs are included because no particular design could make available sufficient data. Secondly, clinical and methodological heterogeneity of the sample population in the form of factors such as the severity of HELLP syndrome, time of CORT administration, and dosage and duration of CORT administration in recruited patients may have impact on overall outcomes. Another factor to compromise the precision of the outcomes may be the handling of missing data. Statistical procedures used to impute measures of dispersal may also have slight impact as some study reports lacked such data.

## CONCLUSIONS

Corticosteroid administration to HELLP syndrome patients provides benefits in improving disease markers. Changes in platelet count and LDH levels after CORT therapy are found to be negatively associated with baseline platelet count, and also with intensive care duration in CORT-treated HELLP syndrome patients. Intensive care duration was, however, positively associated with nulliparity and percentage of patients with comorbidities. Incidence of cesarean delivery was negatively associated with gestation age, but not with any of the biological markers or their changes after CORT therapy. These results favor the use of high-dose CORT therapy to HELLP syndrome patients with greater reductions in platelet count.

## Supplementary Material

Supplemental Digital Content

## References

[1] Sibai BM Diagnosis, controversies, and management of the syndrome of hemolysis, elevated liver enzymes, and low platelet count. *Obstet Gynecol* 2004; 103:981–991. 15121574 10.1097/01.AOG.0000126245.35811.2a

[2] Barton JR Sibai BM Diagnosis and management of hemolysis, elevated liver enzymes, and low platelets syndrome. *Clin Perinatol* 2004; 31:807–833. 15519429 10.1016/j.clp.2004.06.008

[3] Martin JN Jr Rose CH Briery CM Understanding and managing HELLP syndrome: the integral role of aggressive glucocorticoids for mother and child. *Am J Obstet Gynecol* 2006; 195:914–934. 16631593 10.1016/j.ajog.2005.08.044

[4] Vigil-De Gracia P HELLP syndrome. *Ginecol Obstet Mex* 2015; 83:48–57. 26016316

[5] Haram K Svendsen E Abildgaard U The HELLP syndrome: clinical issues and management. A review. *BMC Pregnancy Childbirth* 2009; 9:8. 19245695 10.1186/1471-2393-9-8 PMC2654858

[6] Woudstra DM Chandra S Hofmeyr GJ Corticosteroids for HELLP (hemolysis, elevated liver enzymes, low platelets) syndrome in pregnancy. *Cochrane Database Syst Rev* 2010; CD008148. 20824872 10.1002/14651858.CD008148.pub2 PMC4171033

[7] Mao M Chen C Corticosteroid therapy for management of Hemolysis, Elevated Liver Enzymes, and Low Platelet Count (HELLP) syndrome: a meta-analysis. *Med Sci Monit* 2015; 21:3777–3783. 26633822 10.12659/MSM.895220 PMC4672720

[8] Moher D Liberati A Tetzlaff J The PRISMA Group. Preferred reporting items for systematic reviews and meta-analyses: the PRISMA statement. *PLoS Med* 2009; 6:e1000097. 19621072 10.1371/journal.pmed.1000097 PMC2707599

[9] Higgins JPT Green S (editors). Cochrane Handbook for Systematic Reviews of Interventions Version 5.1.0 [updated March 2011]. http://handbook.cochrane.org/.

[10] Dreyfus M Tissier I Ndocko MA Corticosteroid therapy for conservative management in marginally-viable pregnancy complicated by HELLP syndrome. *Eur J Obstet Gynecol Reprod Biol* 1999; 85:233–234. 10584642 10.1016/s0301-2115(99)00022-6

[11] Fonseca JE Mendez F Catano C Dexamethasone treatment does not improve the outcome of women with HELLP syndrome: a double-blind, placebo-controlled, randomized clinical trial. *Am J Obstet Gynecol* 2005; 193:1591–1598. 16260197 10.1016/j.ajog.2005.07.037

[12] Isler CM Barrilleaux PS Magann EF A prospective, randomized trial comparing the efficacy of dexamethasone and betamethasone for the treatment of antepartum HELLP (hemolysis, elevated liver enzymes, and low platelet count) syndrome. *Am J Obstet Gynecol* 2001; 184:1332–1337.[discussion 1337-9]. 11408849 10.1067/mob.2001.115051

[13] Isler CM Magann EF Rinehart BK Dexamethasone compared with betamethasone for glucocorticoid treatment of postpartum HELLP syndrome. *Int J Gynaecol Obstet* 2003; 80:291–297. 12628531 10.1016/s0020-7292(02)00394-6

[14] Katz L de Amorim MM Figueiroa JN Postpartum dexamethasone for women with hemolysis, elevated liver enzymes, and low platelets (HELLP) syndrome: a double-blind, placebo-controlled, randomized clinical trial. *Am J Obstet Gynecol* 2008; 198:283 e1-8. 18194800 10.1016/j.ajog.2007.10.797

[15] Magann EF Martin RW Isaacs JD Corticosteroids for the enhancement of fetal lung maturity: impact on the gravida with preeclampsia and the HELLP syndrome. *Aust N Z J Obstet Gynaecol* 1993; 33:127–131. 8216107 10.1111/j.1479-828x.1993.tb02374.x

[16] Magann EF Bass D Chauhan SP Antepartum corticosteroids: disease stabilization in patients with the syndrome of hemolysis, elevated liver enzymes, and low platelets (HELLP). *Am J Obstet Gynecol* 1994a; 171:1148–1153. 7943088 10.1016/0002-9378(94)90054-x

[17] Magann EF Perry KG Jr Meydrech EF Postpartum corticosteroids: accelerated recovery from the syndrome of hemolysis, elevated liver enzymes, and low platelets (HELLP). *Am J Obstet Gynecol* 1994b; 171:1154–1158. 7943089 10.1016/0002-9378(94)90055-8

[18] Martin JN Jr Perry KG Jr Blake PG Better maternal outcomes are achieved with dexamethasone therapy for postpartum HELLP (hemolysis, elevated liver enzymes, and thrombocytopenia) syndrome. *Am J Obstet Gynecol* 1997; 177:1011–1017. 9396884 10.1016/s0002-9378(97)70005-x

[19] Martin JN Jr Thigpen BD Rose CH Maternal benefit of high-dose intravenous corticosteroid therapy for HELLP syndrome. *Am J Obstet Gynecol* 2003; 189:830–834. 14526324 10.1067/s0002-9378(03)00763-4

[20] Mecacci F Carignani L Cioni R Time course of recovery and complications of HELLP syndrome with two different treatments: heparin or dexamethasone. *Thromb Res* 2001; 102:99–105. 11323019 10.1016/s0049-3848(01)00234-1

[21] Nunes F Campos AP Avillez T Corticosteroid therapy for patients with HELLP syndrome (hemolysis, elevated liver enzymes, and low platelet count). *Acta Med Port* 2005; 18:177–182. 16207453

[22] O’Brien JM Milligan DA Barton JR Impact of high-dose corticosteroid therapy for patients with HELLP (hemolysis, elevated liver enzymes, and low platelet count) syndrome. *Am J Obstet Gynecol* 2000; 183:921–924. 11035338 10.1067/mob.2000.108869

[23] O’Brien JM Shumate SA Satchwell SL Maternal benefit of corticosteroid therapy in patients with HELLP (hemolysis, elevated liver enzymes, and low platelet count) syndrome: impact on the rate of regional anesthesia. *Am J Obstet Gynecol* 2002; 186:475–479. 11904610 10.1067/mob.2002.121074

[24] Ozer A Kanat-Pektas M Ozer S The effects of betamethasone treatment on clinical and laboratory features of pregnant women with HELLP syndrome. *Arch Gynecol Obstet* 2009; 280:65–70. 19089438 10.1007/s00404-008-0865-3

[25] Tompkins MJ Thiagarajah S HELLP (hemolysis, elevated liver enzymes, and low platelet count) syndrome: the benefit of corticosteroids. *Am J Obstet Gynecol* 1999; 181:304–309. 10454673 10.1016/s0002-9378(99)70552-1

[26] van Runnard Heimel PJ Huisjes AJ Franx A A randomised placebo-controlled trial of prolonged prednisolone administration to patients with HELLP syndrome remote from term. *Eur J Obstet Gynecol Reprod Biol* 2006; 128:187–193. 16412552 10.1016/j.ejogrb.2005.11.041

[27] Varol F Aydin T Gücer F HELLP syndrome and postpartum corticosteroids. *Int J Gynaecol Obstet* 2001; 73:157–159. 11336737 10.1016/s0020-7292(00)00371-4

[28] Vigil-De Gracia P García-Cáceres E Dexamethasone in the post-partum treatment of HELLP syndrome. *Int J Gynaecol Obstet* 1997; 59:217–221. 9486510 10.1016/s0020-7292(97)00214-2

[29] Vigil-De Gracia P Addition of platelet transfusions to corticosteroids does not increase the recovery of severe HELLP syndrome. *Eur J Obstet Gynecol Reprod Biol* 2006; 128:194–198. 16388885 10.1016/j.ejogrb.2005.11.038

[30] Wallace K Martin JN Jr Tam Tam K Seeking the mechanism(s) of action for corticosteroids in HELLP syndrome: SMASH study. *Am J Obstet Gynecol* 2013; 208:380 e1-8. 23380266 10.1016/j.ajog.2013.01.049

[31] Yalcin OT Sener T Hassa H Effects of postpartum corticosteroids in patients with HELLP syndrome. *Int J Gynaecol Obstet* 1998; 61:141–148. 9639218 10.1016/s0020-7292(98)00036-8

[32] Isler CM Rinehart BK Terrone DA Maternal mortality associated with HELLP (hemolysis, elevated liver enzymes, and low platelets) syndrome. *Am J Obstet Gynecol* 1999; 181:924–928. 10521755 10.1016/s0002-9378(99)70343-1

[33] Katz L Mr Amorim M Souza A Sr Risk factors for cesarean section in women with severe preeclampsia. *Pregnancy Hypertens* 2015; 5:68.

[34] Haddad B Barton JR Livingston JC HELLP (hemolysis, elevated liver enzymes, and low platelet count) syndrome versus severe preeclampsia: onset at <or = 280 weeks’ gestation. *Am J Obstet Gynecol* 2000; 183:1475–1479. 11120513 10.1067/mob.2000.106975

[35] Haddad B Barton JR Livingston JC Risk factors for adverse maternal outcomes among women with HELLP (hemolysis, elevated liver enzymes, and low platelet count) syndrome. *Am J Obstet Gynecol* 2000b; 183:444–448. 10942484 10.1067/mob.2000.105915

[36] Bezircioglu I Baloglu A Cetinkaya B Do clinical and laboratory parameters effect maternal and fetal outcomes in pregnancies complicated with hemolysis, elevated liver enzymes, and low platelet count syndrome? *J Turk Ger Gynecol Assoc* 2012; 13:1–7. 24627667 10.5152/jtgga.2011.68 PMC3940217

