# Supplementary material for: Prognostic Factors of the Efficacy of High-dose Corticosteroid Therapy in Hemolysis, Elevated Liver Enzymes, and Low Platelet Count Syndrome During Pregnancy: A Meta-analysis
Source: Medicine (Baltimore). 2016 Apr 1;95(13):e3203. doi: 10.1097/MD.0000000000003203 (PMC4998544; doi:10.1097/MD.0000000000003203)
Supplement: Supplemental Digital Content [file medi-95-e3203-s001.doc]

**Supplementary Information**

| **Study** | **n** | **Design** | **CORT admn.** | **% Ces.** | **Total dose CORT** | **Age** | **sd** | **HELLP Class (%)** | | | **Gest. age** | **sd** | **% Nulli-parous** | **≥1 parity** | **Baseline values of markers** | | | | | | | | **MBP** | **SD** | **UO** | **SD** | **% BT** | **ICS** | **SD** |
| --- | --- | --- | --- | --- | --- | --- | --- | --- | --- | --- | --- | --- | --- | --- | --- | --- | --- | --- | --- | --- | --- | --- | --- | --- | --- | --- | --- | --- | --- |
|  |  |  |  |  |  |  |  | **1** | **2** | **3** |  |  |  |  | **Plate.** | **SD** | **AST** | **SD** | **ALT** | **SD** | **LDH** | **SD** |  |  |  |  |  |  |  |
| Dreyfus 1999 | 6 | RET | AP | 67 | 132 |  |  |  |  |  | 27 | 1 |  |  | 78.3 | 15 | 364.2 | 563.7 |  |  |  |  |  |  |  |  |  |  |  |
| Fonseca 2005 | 66 | RCT | AP |  |  | 24.5 | 7 | 42.4 | 57.6 | 0 | 33.8 | 4.5 |  |  | 58.4 | 21 | 573 | 621 | 281 | 300 | 2124 | 1849 | 127.7 | 18.2 |  |  | 95 |  |  |
| Isler 2001 dexa | 19 | RCT | AP | 52.6 |  | 24.9 | 5.5 |  |  |  | 30.9 | 4.9 | 14 | 5 | 73.6 | 20.3 | 143.1 | 133.5 |  |  | 1447 | 977.3 | 116.3 | 9.2 | 89.2 | 45 | 10.5 |  |  |
| Isler 2001 beta | 21 | RCT | AP | 33.3 |  | 26.5 | 7.8 |  |  |  | 31.6 | 5 | 9 | 12 | 81.1 | 17 | 129.9 | 99.7 |  |  | 1217.9 | 99.7 | 125.4 | 20.5 | 88.1 | 39.4 | 4.76 |  |  |
| Isler 2003 dexa | 18 | RCT | PP |  |  | 24.8 | 5.6 |  |  |  | 31.1 | 4.6 | 12 | 6 | 72.7 | 20.6 | 176.9 | 161.4 |  |  | 1831.7 | 1141 | 114.2 | 9.6 | 86.9 | 45.2 |  | 1.5 | 0.54 |
| Isler 2003 beta | 18 | RCT | PP |  |  | 24.1 | 7.1 |  |  |  | 32.8 | 4.8 | 11 | 7 | 81 | 17.9 | 101.9 | 73 |  |  | 1193.6 | 496.4 | 111.2 | 8 | 76.2 | 51.2 |  | 1.75 | 0.37 |
| Katz 2008 | 56 | RCT | PP | 75 | 80 | 25.5 | 5.7 |  |  |  | 34.2 | 4.2 | 59 | 41 | 91.2 | 28 | 154.9 | 226 |  |  | 1101 | 1500 | 141.6 | 24.3 | 83 | 23.4 | 28.6 | 10.2 | 5.3 |
| Magann 1993 | 27 | RET | AP | 74 |  | 23.4 | 5.5 | 22.2 | 37 | 40.7 | 28 | 4.8 |  |  |  |  |  |  |  |  |  |  |  |  |  |  | 30 | 29.8 | 50.6 |
| Magann 1994a | 12 | RCT | AP |  |  | 23.7 | 7.5 |  |  |  | 30.7 | 4.9 | 0 | 100 | 69.3 | 23 | 232 | 355 | 144 | 150 | 1673 | 1947 | 124.8 | 14 | 48 | 4 |  |  |  |
| Magann 1994b | 20 | RCT | PP |  | 30 | 24.4 | 6.6 |  |  |  | 33.7 | 3.1 |  |  | 66 | 13 |  |  | 170 | 125 | 1700 | 1665 | 123.7 | 16.5 | 45 | 23.4 |  |  |  |
| Martin 1997 | 43 | RET | PP | 62 | 150 | 23.6 | 6 | 40 | 60 | 0 |  |  |  |  | 86 | 30.3 |  |  |  |  | 2000 | 1480 |  |  |  |  |  | 4.3 | 1.6 |
| Martin 2003 | 228 | RET | PeriP | 55 |  | 23.2 | 6 |  |  |  | 32 | 3.8 | 66 | 34 | 115 | 62 | 150 | 368 |  |  | 1556 | 1453 |  |  |  |  |  |  |  |
| Meccai 2001 | 12 | RET | PP | 91.7 |  | 33.5 | 4 | 42 | 42 | 16 | 29.1 | 3.5 | 58 | 42 | 61.4 | 26 |  |  |  |  | 1471 | 665.2 | 120 | 15 |  |  | 41.7 | 1.5 | 2.25 |
| Nunes 2005 | 35 | RET | PeriP | 48 | 95 | 26.8 | 5.5 |  |  |  | 31.8 | 4.1 | 62 | 38 | 64 | 26.4 | 267 | 263 | 253 | 200 | 790 | 502 |  |  |  |  |  |  |  |
| O'Brien 2000 | 11 | RET | AP | 64 | 80 | 29 | 5 |  |  |  | 32 | 5 |  |  | 75 | 51 | 180 | 122 |  |  | 1421 | 610 | 102 | 40.7 |  |  |  |  |  |
| O'Brien 2002 | 46 | RET | AP | 46 |  | 27.4 | 6 |  |  |  | 32.4 | 3.4 | 78 | 22 | 92 | 48 | 188 | 181 |  |  | 1402 | 823 |  |  |  |  |  |  |  |
| Ozer 2009 | 30 | RCT | AP | 76.7 |  | 28.5 | 7.3 | 17 | 57 | 26 | 32.4 | 4.5 | 50 |  | 79.4 | 34.5 | 248.1 | 288.2 | 205 | 223 | 1331.2 | 620.6 | 116.3 | 14.4 |  |  | 13.3 | 4.14 | 1.97 |
| Thomkins 1999 bet. 12h | 44 | RET | AP | 94.6 | 24 | 25 |  |  |  |  | 29.7 |  |  |  |  |  |  |  |  |  |  |  |  |  |  |  |  |  |  |
| Thomkins 1999 bet. 24h | 27 | RET | AP | 94.6 | 24 | 25 |  |  |  |  | 29.7 |  |  |  |  |  |  |  |  |  |  |  |  |  |  |  |  |  |  |
| Thomkins 1999 dexa. | 12 | RET | AP | 94.6 | 24 | 25 |  |  |  |  | 29.7 |  |  |  |  |  |  |  |  |  |  |  |  |  |  |  |  |  |  |
| van Runnard Heimel 2006 | 15 | RET | PeriP | 100 |  | 31.6 | 2.5 |  |  |  | 27.4 | 1.4 | 93 | 7 | 105 | 38 | 154 | 241 | 197 | 212 | 1410.5 | 1132 | 110.3 | 17.4 |  |  | 26.6 | 29.4 | 11.9 |
| Varol 2001 | 9 | RET | PP |  | 30 | 26.5 | 5.6 |  |  |  | 33.5 | 3.3 |  |  | 60 |  |  |  |  |  |  |  |  |  |  |  |  | 7.7 | 3 |
| Vigil-De Gracia 1997 | 17 | RCT | PP | 76.4 | 30 | 25 | 5.3 |  |  |  | 32.8 | 3.42 |  |  | 50.8 | 54.5 | 472.3 | 192 | 231 | 101 | 792 | 300 | 138.5 | 22.1 |  |  |  |  |  |
| Vigil-De Gracia 2006 | 26 | RET | AP/PP | 53 |  | 29.4 | 7 | 100 |  |  | 34.4 | 3 |  |  | 36 | 10.5 | 630 | 584 | 403 | 202 | 2400 | 1900 |  |  |  |  |  | 6.2 | 2 |
| Wallace 2013 | 17 | Non-RCT | PeriP | 71 |  | 24 | 1.5 | 35 | 47 | 18 | 31.9 | 1.2 | 6 | 11 | 80.6 | 5.57 | 195.5 | 43.24 |  |  | 1737 | 743 |  |  |  |  |  |  |  |
| Yalcin 1998 | 15 | RCT | PP | 46.7 | 30 | 26.1 | 8.9 |  |  |  | 35.1 | 2.9 | 67 | 33 | 77.5 | 23.5 | 127.2 | 28.1 | 133 | 16.1 |  |  | 130.2 | 13.8 | 19.2 | 10.1 | 40 | 6 | 4.1 |

Abbreviations: Admn., administration, ALT, alanine aminotransferase (IU/L); AST, aspartate aminotransferase (IU/L); BT, blood transfusion; Ces. Cesarean delivery, CORT, corticosteroid; Gest., gestation ; ICS, intensive care stay (days); LDH, lactic dehydrogenase (IU/L); MBP, mean blood pressure; n, number of patients; Plate., platelets; UO, urinary output (ml/h);

**Factors identified in the included studies**

| **Study** | **Factors identified** |
| --- | --- |
| Fonseca et al 2005 | - The duration of hospitalization was shorter among women who received dexamethasone therapy. - Multivariate analysis showed that a longer duration of hospitalization was associated with a lower urinary output (130 mL/h) and higher LDH levels. - Among 49 patients with HELLP 1 (28 patients with placebo and 21 patients with dexamethasone therapy), the conditional probability of platelet recovery was higher in those patients who received dexamethasone therapy, even after adjustment for potential confounders. |
| Katz et al 2008 | The adjustment in linear mixed models for correlated data indicated that dexamethasone therapy had no significant effect on platelet count, log LDH, diuresis, AST, and hemoglobin |
| Magann et al 1994a | Stepwise discriminant analysis revealed rates of change for urinary output and LDH to be factors significantly affected because of the choice of therapy. However, by excluding urinary output in the step wise analysis, platelets, alanine aminotransferase, and LDH were found to be significantly influenced by treatment choice |

**Literature search strategy for PubMed**

1. ("haemolysis"[All Fields] OR "hemolysis"[MeSH Terms] OR "hemolysis"[All Fields]) AND elevated[All Fields] AND ("liver"[MeSH Terms] OR "liver"[All Fields]) AND ("enzymology"[Subheading] OR "enzymology"[All Fields] OR "enzymes"[All Fields] OR "enzymes"[MeSH Terms]) AND low[All Fields] AND ("platelet count"[MeSH Terms] OR ("platelet"[All Fields] AND "count"[All Fields]) OR "platelet count"[All Fields]) AND ("syndrome"[MeSH Terms] OR "syndrome"[All Fields]) AND ("adrenal cortex hormones"[MeSH Terms] OR ("adrenal"[All Fields] AND "cortex"[All Fields] AND "hormones"[All Fields]) OR "adrenal cortex hormones"[All Fields] OR "corticosteroid"[All Fields])

2. ("hellp syndrome"[MeSH Terms] OR ("hellp"[All Fields] AND "syndrome"[All Fields]) OR "hellp syndrome"[All Fields]) AND ("adrenal cortex hormones"[MeSH Terms] OR ("adrenal"[All Fields] AND "cortex"[All Fields] AND "hormones"[All Fields]) OR "adrenal cortex hormones"[All Fields] OR "corticosteroid"[All Fields])

3. ("hellp syndrome"[MeSH Terms] OR ("hellp"[All Fields] AND "syndrome"[All Fields]) OR "hellp syndrome"[All Fields] OR "hellp"[All Fields]) AND ("platelet count"[MeSH Terms] OR ("platelet"[All Fields] AND "count"[All Fields]) OR "platelet count"[All Fields]) AND ("adrenal cortex hormones"[MeSH Terms] OR ("adrenal"[All Fields] AND "cortex"[All Fields] AND "hormones"[All Fields]) OR "adrenal cortex hormones"[All Fields] OR "corticosteroid"[All Fields])

4. ("hellp syndrome"[MeSH Terms] OR ("hellp"[All Fields] AND "syndrome"[All Fields]) OR "hellp syndrome"[All Fields] OR "hellp"[All Fields]) AND ("aspartate aminotransferases"[MeSH Terms] OR ("aspartate"[All Fields] AND "aminotransferases"[All Fields]) OR "aspartate aminotransferases"[All Fields] OR ("aspartate"[All Fields] AND "aminotransferase"[All Fields]) OR "aspartate aminotransferase"[All Fields]) AND ("adrenal cortex hormones"[MeSH Terms] OR ("adrenal"[All Fields] AND "cortex"[All Fields] AND "hormones"[All Fields]) OR "adrenal cortex hormones"[All Fields] OR "corticosteroid"[All Fields])

5. ("hellp syndrome"[MeSH Terms] OR ("hellp"[All Fields] AND "syndrome"[All Fields]) OR "hellp syndrome"[All Fields] OR "hellp"[All Fields]) AND ("alanine transaminase"[MeSH Terms] OR ("alanine"[All Fields] AND "transaminase"[All Fields]) OR "alanine transaminase"[All Fields] OR ("alanine"[All Fields] AND "aminotransferase"[All Fields]) OR "alanine aminotransferase"[All Fields]) AND ("adrenal cortex hormones"[MeSH Terms] OR ("adrenal"[All Fields] AND "cortex"[All Fields] AND "hormones"[All Fields]) OR "adrenal cortex hormones"[All Fields] OR "corticosteroid"[All Fields])

6. ("hellp syndrome"[MeSH Terms] OR ("hellp"[All Fields] AND "syndrome"[All Fields]) OR "hellp syndrome"[All Fields] OR "hellp"[All Fields]) AND lactic[All Fields] AND ("oxidoreductases"[MeSH Terms] OR "oxidoreductases"[All Fields] OR "dehydrogenase"[All Fields]) AND ("adrenal cortex hormones"[MeSH Terms] OR ("adrenal"[All Fields] AND "cortex"[All Fields] AND "hormones"[All Fields]) OR "adrenal cortex hormones"[All Fields] OR "corticosteroid"[All Fields])

7. ("hellp syndrome"[MeSH Terms] OR ("hellp"[All Fields] AND "syndrome"[All Fields]) OR "hellp syndrome"[All Fields]) AND ("dexamethasone"[MeSH Terms] OR "dexamethasone"[All Fields])

8. ("hellp syndrome"[MeSH Terms] OR ("hellp"[All Fields] AND "syndrome"[All Fields]) OR "hellp syndrome"[All Fields]) AND ("betamethasone"[MeSH Terms] OR "betamethasone"[All Fields])

9. ("hellp syndrome"[MeSH Terms] OR ("hellp"[All Fields] AND "syndrome"[All Fields]) OR "hellp syndrome"[All Fields]) AND ("prednisolone"[MeSH Terms] OR "prednisolone"[All Fields])

10. ("hellp syndrome"[MeSH Terms] OR ("hellp"[All Fields] AND "syndrome"[All Fields]) OR "hellp syndrome"[All Fields]) AND ("adrenal cortex hormones"[MeSH Terms] OR ("adrenal"[All Fields] AND "cortex"[All Fields] AND "hormones"[All Fields]) OR "adrenal cortex hormones"[All Fields] OR "corticosteroid"[All Fields]) AND ("postpartum period"[MeSH Terms] OR ("postpartum"[All Fields] AND "period"[All Fields]) OR "postpartum period"[All Fields] OR "postpartum"[All Fields])

11. ("hellp syndrome"[MeSH Terms] OR ("hellp"[All Fields] AND "syndrome"[All Fields]) OR "hellp syndrome"[All Fields]) AND ("adrenal cortex hormones"[MeSH Terms] OR ("adrenal"[All Fields] AND "cortex"[All Fields] AND "hormones"[All Fields]) OR "adrenal cortex hormones"[All Fields] OR "corticosteroid"[All Fields]) AND antepartum[All Fields]

12. ("hellp syndrome"[MeSH Terms] OR ("hellp"[All Fields] AND "syndrome"[All Fields]) OR "hellp syndrome"[All Fields]) AND ("adrenal cortex hormones"[MeSH Terms] OR ("adrenal"[All Fields] AND "cortex"[All Fields] AND "hormones"[All Fields]) OR "adrenal cortex hormones"[All Fields] OR "corticosteroid"[All Fields]) AND ("peripartum period"[MeSH Terms] OR ("peripartum"[All Fields] AND "period"[All Fields]) OR "peripartum period"[All Fields] OR "peripartum"[All Fields])

S1 Figure: Forest graph of the meta-analysis of change from baseline in AST levels.


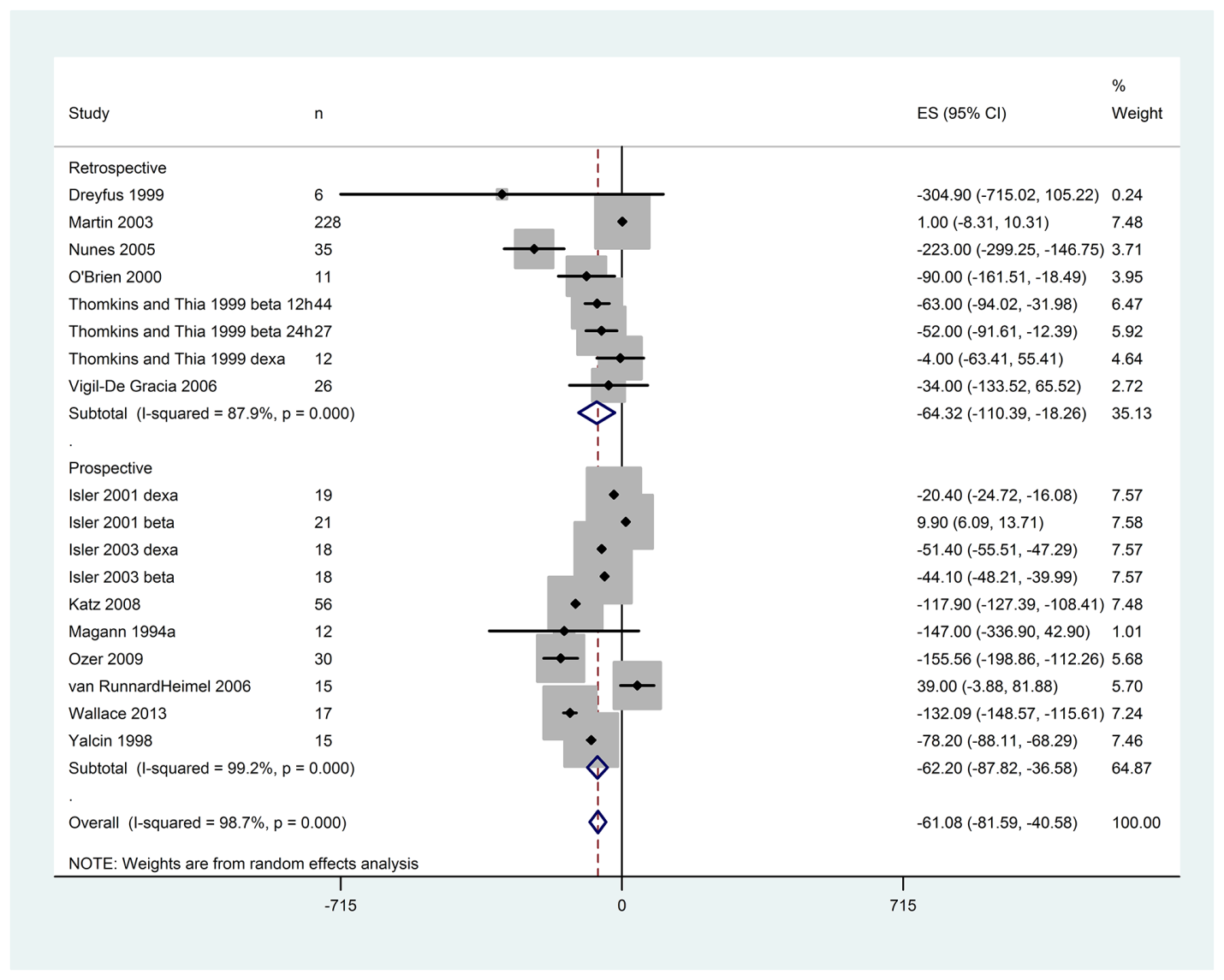


S2 Figure: Forest graph of the meta-analysis of change from baseline in ALT levels.


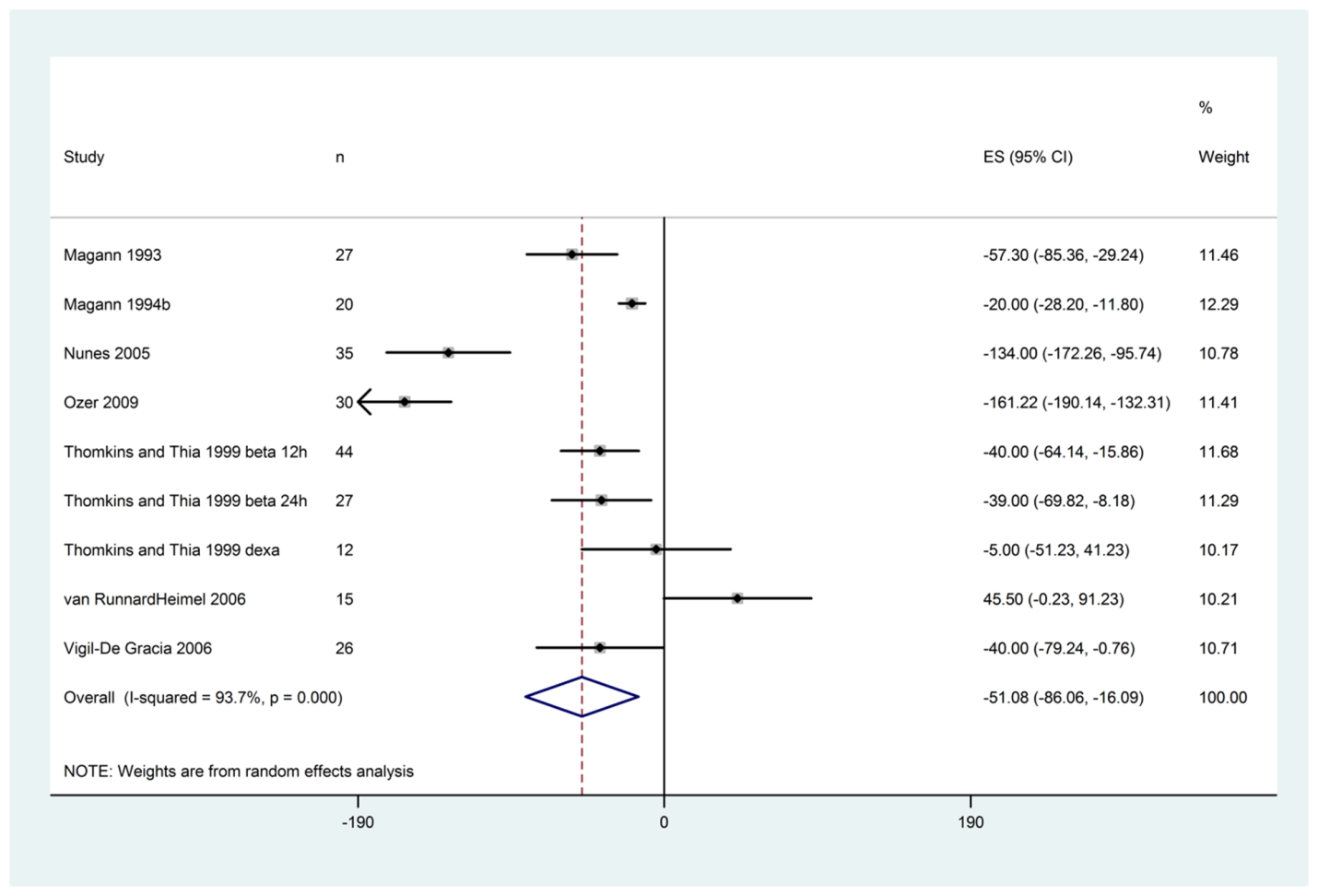


S3 Figure: Forest graph of the meta-analysis of change from baseline in LDH levels


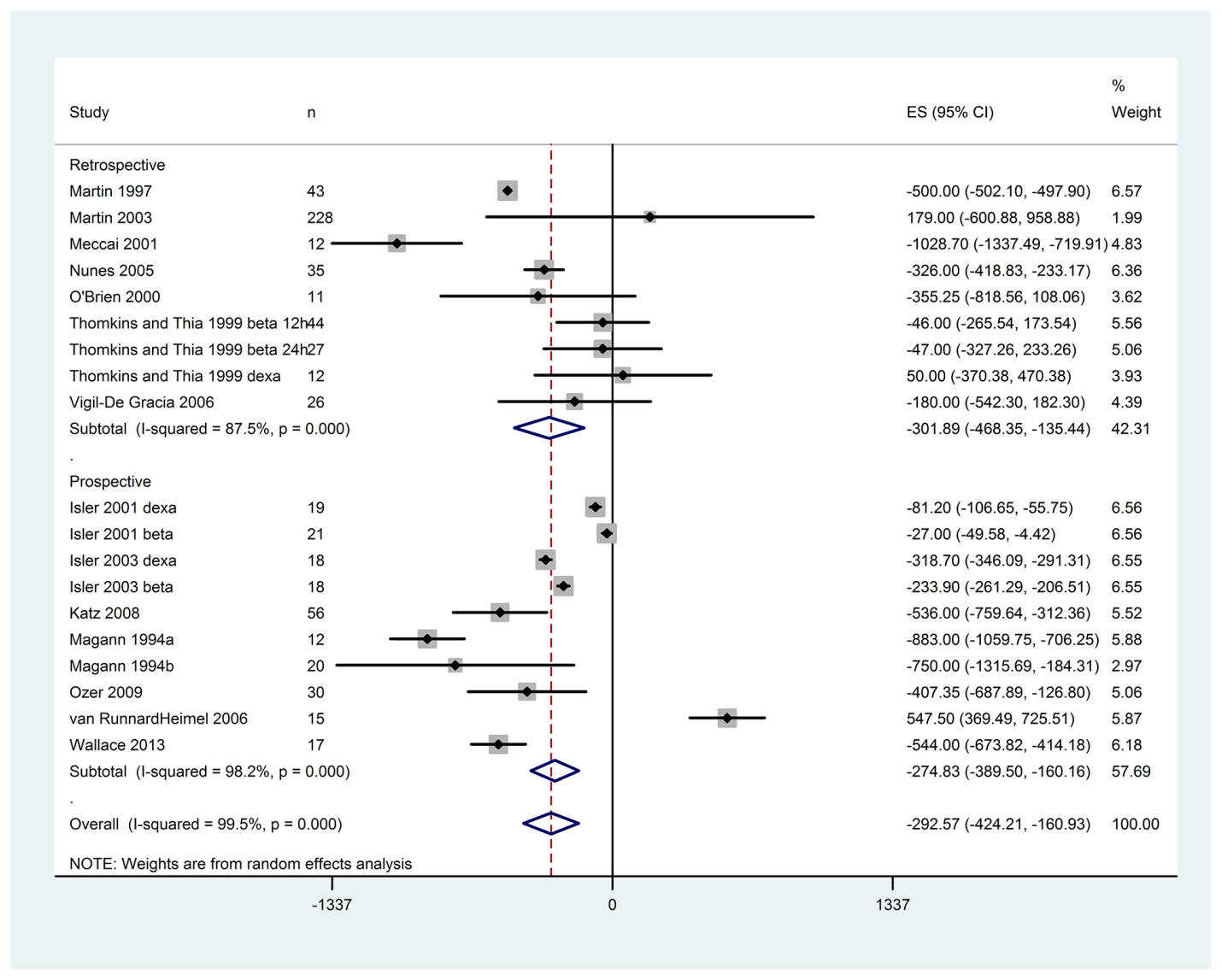


S4 Figure: Forest graph of the meta-analysis of change from baseline in mean blood pressure


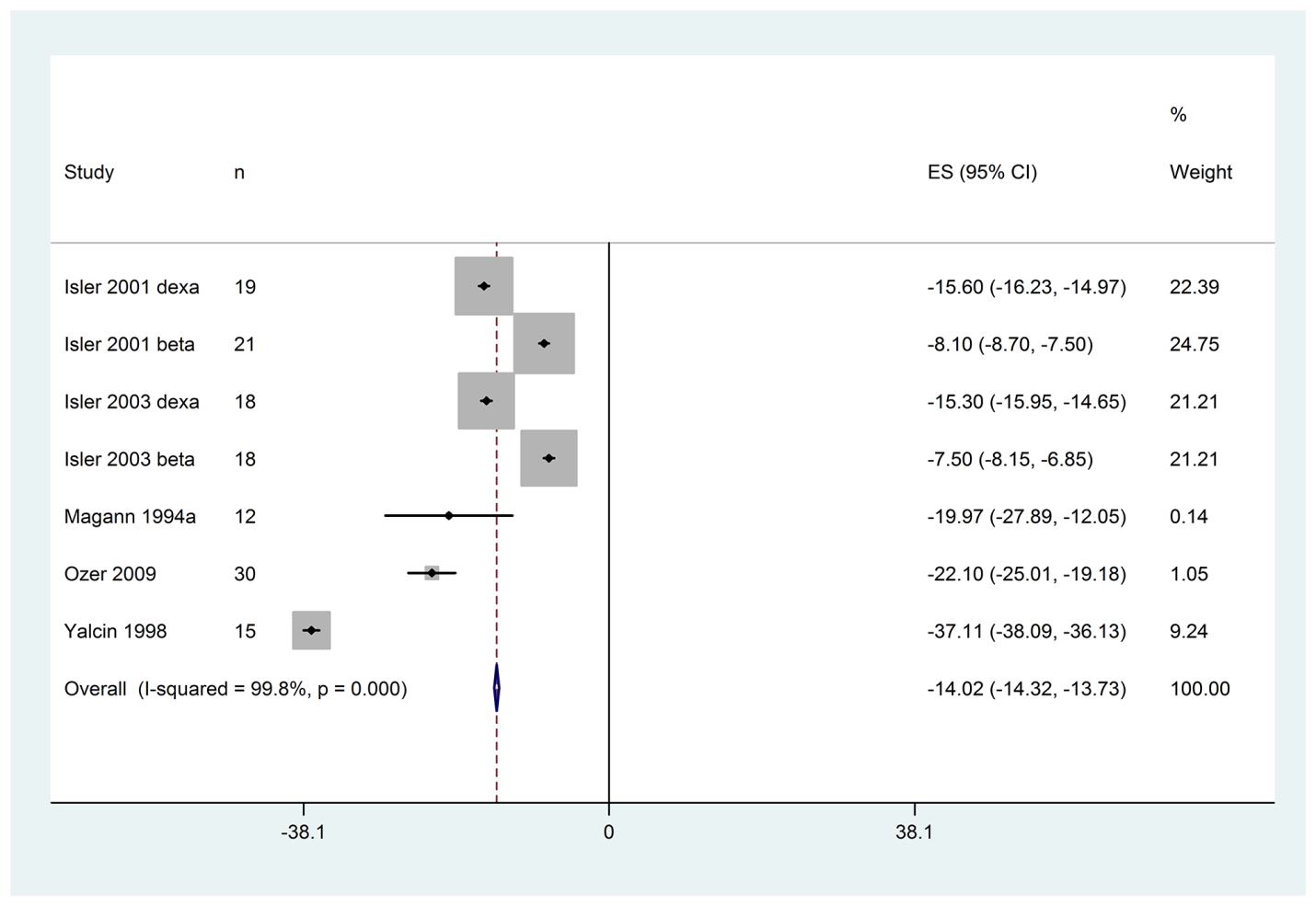


S5 Figure: Forest graph of the meta-analysis of change from baseline in urinary output


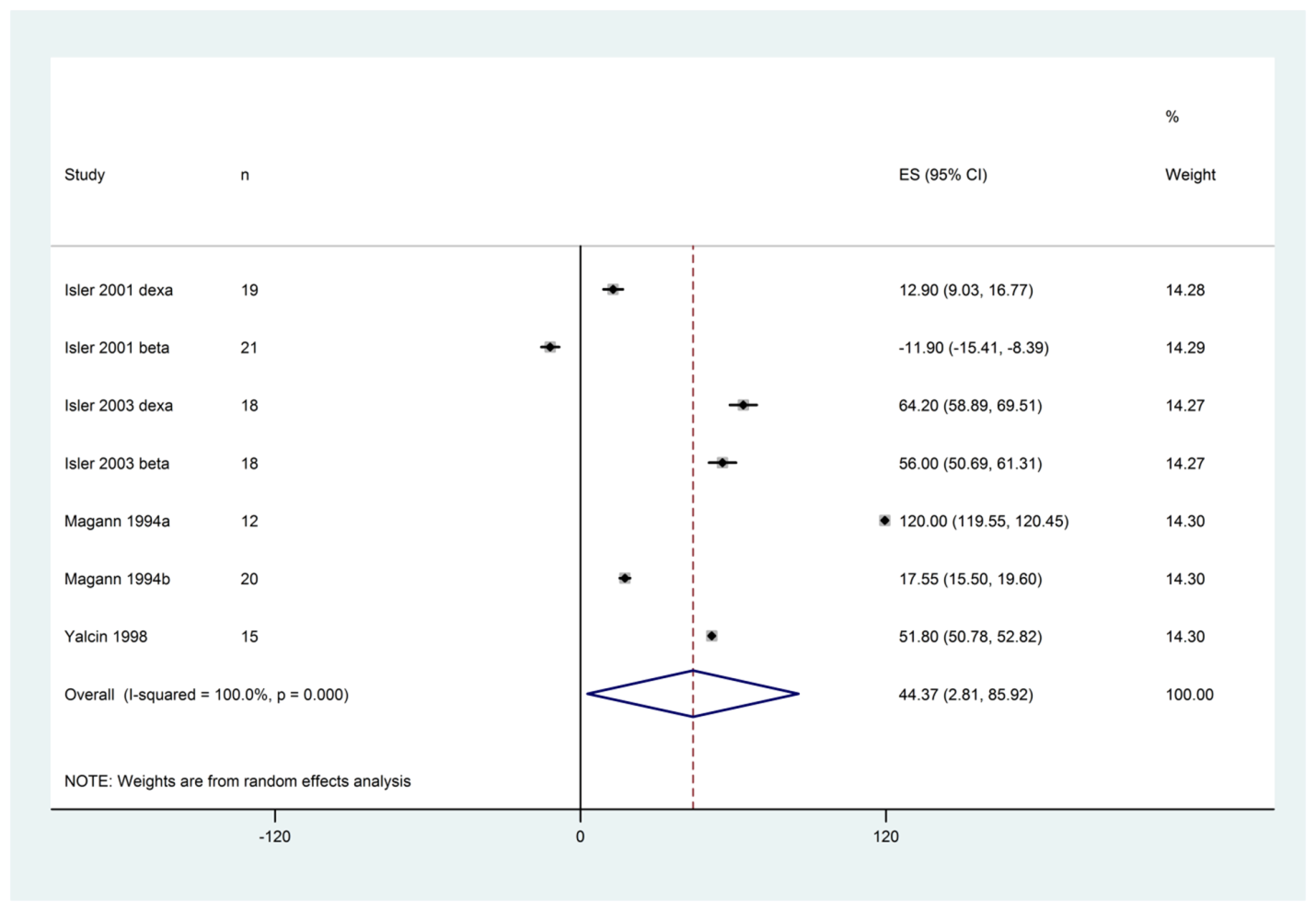


| **S1 Table: Predictors and prognostic factors of the change from baseline in platelet count in CORT treated patients** | | | | |
| --- | --- | --- | --- | --- |
|  | **Coefficient [95% confidence interval]** | **p** | **Studies / datasets** | **tau^2^** |
| Number of study patients | −0.228 [−545, 0.087] | 0.148 | 25 | 1046 |
| Mother’s age (years) | 3.726 [−2.011, 9.464] | 0.192 | 24 | 1060 |
| Gestation age (weeks) | 2.055 [−5.164, 9.275] | 0.561 | 24 | 1166 |
| Nulliparous percentage | 0.150 [−0.568, 0.868] | 0.657 | 14 | 1367 |
| CORT total dose (mg) | 0.166 [−0.362, 0.694] | 0.499 | 12 | 1186 |
| Baseline platelet count (x10^9^) | −0.893 [−1.945, 0.158] | **0.092** | 21 | 1171 |
| Baseline AST levels (IU/L) | 0.054 [−0.101, 0.209] | 0.468 | 17 | 1376 |
| Baseline ALT levels (IU/L) | 0.124 [−0.526, 0.774] | 0.658 | 8 | 1319 |
| Baseline LDH levels (IU/L) | −0.053 [−0.098, 0.007] | **0.027** | 18 | 1017 |
| Baseline mean blood pressure | 1.353 [−0.666, 3.372] | 0.170 | 14 | 1308 |
| Baseline urinary output (ml/h) | 0.174 [−1.205, 1.554] | 0.768 | 8 | 1517 |
| Blood transfusion (% required) | −0.181 [−1.553, 1.190] | 0.763 | 9 | 1913 |
| Cesarean delivery percentage | 0.404 [−0.495, 1.303] | 0.357 | 19 | 1220 |
| Intensive care stay (days) | −0.948 [−3.401, 1.504] | 0.399 | 10 | 1177 |
| % with comorbidities | −0.572, [−2.331, 1.186] | 0.509 | 27 | 1028 |
| Abbreviations: ALT, alanine aminotransferase; AST, aspartate aminotransferase; CI, confidence interval; CORT, corticosteroid; ICU, intensive care unit, IU, international unit; L, liter; LDH, lactic dehydrogenase; mg, milligram, ml, milliliter, n, number of included studies/datasets; SE, standard error; UO, urinary output. | | | | |

| **S2 Table: Predictors and prognostic factors of the change from baseline in AST levels in CORT treated patients** | | | | |
| --- | --- | --- | --- | --- |
|  | **Coefficient [95% confidence interval]** | **P** | **Studies / datasets** | **tau^2^** |
| Number of study patients | 0.231 [−0.431, 0.891] | 0.471 | 18 | 3868 |
| Mother’s age (years) | 1.884 [−14.29, 18.059] | 0.807 | 17 | 3968 |
| Gestation age (weeks) | −12.08 [−29.23, 5.071] | 0.155 | 18 | 3124 |
| Nulliparous percentage | 0.083 [−1.651, 1.817] | 0.917 | 12 | 5808 |
| CORT total dose (mg) | −1.399 [−2.384, −0.415] | **0.013** | 8 | 405 |
| Baseline platelet count (x10^9^) | 1.527 [−0.870, 3.924] | 0.192 | 15 | 4078 |
| Baseline AST levels (IU/L) | −0.141 [−0.517, 0.239] | 0.428 | 15 | 4504 |
| Baseline ALT levels (IU/L) | 0.096 [−1.339, 1.532] | 0.861 | 6 | 10488 |
| Baseline LDH levels (IU/L) | 0.0613 [−0.068, 0.191] | 0.320 | 13 | 4984 |
| Baseline mean blood pressure | −1.550 [−5.687, 2.587] | 0.413 | 10 | 3379 |
| Blood transfusion (% required) | −1.281 [−8.858, 6.294] | 0.663 | 6 | 6323 |
| Baseline urinary output (ml/h) | 0.703 [−1.199, 2.606] | 0.386 | 7 | 2063 |
| Cesarean delivery percentage | 0.335 [−1.728, 2.399] | 0.731 | 15 | 4901 |
| Intensive care stay (days) | 3.757 [−2.294, 9.809] | 0.171 | 7 | 2736 |
| % with comorbidities | 1.363 [−2.342, 5.069] | 0.450 | 20 | 3128 |
| Abbreviations: ALT, alanine aminotransferase; AST, aspartate aminotransferase; CI, confidence interval; CORT, corticosteroid; ICU, intensive care unit, IU, international unit; L, liter; LDH, lactic dehydrogenase; mg, milligram, ml, milliliter, n, number of included studies/datase ts; SE, standard error; UO, urinary output. | | | | |

| **S3 Table: Predictors and prognostic factors of the change from baseline in ALT levels in CORT treated patients** | | | | |
| --- | --- | --- | --- | --- |
|  | **Coefficient [95% confidence interval]** | **P** | **Studies / datasets** | **tau^2^** |
| Number of study patients | −3.578 [−8.321, 1.164] | 0.118 | 9 | 2792 |
| Mother’s age (years) | 1.651 [−19.01, 22.308] | 0.855 | 9 | 4113 |
| Gestation age (weeks) | −10.23 [−31.23, 10.77] | 0.287 | 9 | 3474 |
| Nulliparous percentage | 0.083 [−1.651, 1.817] | 0.917 | 12 | 5808 |
| CORT total dose (mg) | −1.509 [−2.692, −0.327] | **0.027** | 5 | 188 |
| Baseline platelet count (x10^9^) | 0.901 [−5.122, 6.924] | 0.666 | 5 | 8516 |
| Baseline AST levels (IU/L) | Insufficient data (n=4) | | | |
| Baseline ALT levels (IU/L) | −0.041 [−1.713, 1.631] | 0.943 | 5 | 9164 |
| Baseline LDH levels (IU/L) | 0.063 [−0.175, 0.301] | 0.462 | 5 | 7298 |
| Baseline mean blood pressure | Insufficient data (n=3) | | | |
| Baseline urinary output (ml/h) | Insufficient data (n=3) | | | |
| Blood transfusion (% required) | Insufficient data (n=3) | | | |
| Cesarean delivery percentage | 1.976 [−0.666, 4.620] | 0.117 | 8 | 2921 |
| Intensive care stay (days) | Insufficient data (n=4) | | | |
| % with comorbidities | 1.457 [−3.228, 6.142] | 0.500 | 11 | 3524 |
| Abbreviations: ALT, alanine aminotransferase; AST, aspartate aminotransferase; CI, confidence interval; CORT, corticosteroid; ICU, intensive care unit, IU, international unit; L, liter; LDH, lactic dehydrogenase; mg, milligram, ml, milliliter, n, number of included studies/datase ts; SE, standard error; UO, urinary output. | | | | |

| **S4 Table: Predictors and prognostic factors of the change from baseline in LDH levels in CORT treated patients** | | | | |
| --- | --- | --- | --- | --- |
|  | **Coefficient [95% confidence interval]** | **P** | **Studies / datasets** | **tau^2^** |
| Number of study patients | 2.021 [−3.190, 7.232] | 0.425 | 19 | 122602 |
| Mother’s age (years) | 8.544 [−58.39, 75.488] | 0.791 | 19 | 126156 |
| Gestation age (weeks) | −74.37 [−177.93, 29.20] | 0.147 | 18 | 113622 |
| Nulliparous percentage | 0.083 [−1.651, 1.817] | 0.917 | 12 | 5808 |
| CORT total dose (mg) | −2.916 [−4.470, −1.362] | **0.004** | 8 | 0 |
| Baseline platelet count (x10^9^) | 10.833 [−1.245, 22.912] | **0.075** | 16 | 108663 |
| Baseline AST levels (IU/L) | −0.348 [−2.184, 1.488] | 0.684 | 13 | 121248 |
| Baseline ALT levels (IU/L) | 1.603 [−5.917, 9.123] | 0.586 | 6 | 285479 |
| Baseline LDH levels (IU/L) | −0.127 [−0.693, 0.439] | 0.638 | 16 | 137493 |
| Baseline mean blood pressure | −16.448 [−46.69, 13.80] | 0.250 | 11 | 170048 |
| Baseline urinary output (ml/h) | 16.61 [5.409, 27.803] | **0.012** | 7 | 23305 |
| Blood transfusion (% required) | −18.474 [−65.55, 28.60] | 0.663 | 6 | 258975 |
| Incidence of cesarean delivery (%) | 1.695 [−8.916, 12.306] | 0.736 | 15 | 129231 |
| Intensive care stay (days) | 35.99 [8.131, 63.866] | **0.020** | 8 | 69857 |
| % with comorbidities | 9.872 [−12.662, 32.41] | 0.371 | 21 | 134923 |
| Abbreviations: ALT, alanine aminotransferase; AST, aspartate aminotransferase; CI, confidence interval; CORT, corticosteroid; ICU, intensive care unit, IU, international unit; L, liter; LDH, lactic dehydrogenase; mg, milligram, ml, milliliter, n, number of included studies/datase ts; SE, standard error; UO, urinary output. | | | | |

| **S5 Table: Predictors and prognostic factors of the cesarean delivery rate in CORT treated HELLP syndrome patients** | | | | |
| --- | --- | --- | --- | --- |
|  | **Coefficient [95% confidence interval]** | **P** | **Studies / datasets** | **tau^2^** |
| Number of study patients | −0.0006 [−0.003, 0.002] | 0.578 | 20 | 0 |
| Mother’s age (years) | 0.002 [−0.048, 0.053] | 0.924 | 19 | 0 |
| Gestation age (weeks) | −0.053 [−0.112, 0.006] | **0.074** | 19 | 0 |
| Nulliparous percentage | 0.0004 [−0.006, 0.007] | 0.899 | 11 | 0 |
| CORT total dose (mg) | −0.002 [−0.005, 0.001] | 0.170 | 10 | 0 |
| Baseline platelet count (x10^9^) | −0.001 [−0.007, 0.006] | 0.804 | 16 | 0 |
| Baseline AST levels (IU/L) | 0.0002 [−0.0008, 0.0012] | 0.660 | 14 | 0 |
| Baseline ALT levels (IU/L) | −0.0004 [−0.003, 0.002] | 0.685 | 6 | 0 |
| Baseline LDH levels (IU/L) | −0.00003 [−0.0003, 0.0003] | 0.986 | 14 | 0 |
| Baseline mean blood pressure | −0.0008 [−0.016, 0.015] | 0.902 | 9 | 0 |
| Baseline urinary output (ml/h) | Insufficient data (n=4) | | | |
| Blood transfusion (% required) | 0.008 [−0.006, 0.022] | 0.205 | 8 | 0 |
| Intensive care stay (days) | 0.0064 [−0.0088, 0.0217] | 0.341 | 8 | 0 |
| % with comorbidities | −0.00017 [−0.018, 0.017] | 0.984 | 21 | 0 |
| Abbreviations: ALT, alanine aminotransferase; AST, aspartate aminotransferase; CI, confidence interval; CORT, corticosteroid; ICU, intensive care unit, IU, international unit; L, liter; LDH, lactic dehydrogenase; mg, milligram, ml, milliliter, n, number of included studies/datase ts; SE, standard error; UO, urinary output. | | | | |

| **S6 Table: Predictors and prognostic factors of the intensive care (days) in CORT treated HELLP syndrome patients** | | | | |
| --- | --- | --- | --- | --- |
|  | **Coefficient [95% confidence interval]** | **P** | **Studies / datasets** | **tau^2^** |
| Number of study patients | 0.0035 [−0.465, 0.472] | 0.987 | 11 | 74 |
| Mother’s age (years) | 0.445 [−1.594, 2.483] | 0.633 | 11 | 68 |
| Gestation age (weeks) | −1.912 [−4.401, 0.576] | 0.114 | 10 | 63 |
| Nulliparous percentage | 0.240 [−0.012, 0.493] | **0.059** | 7 | 45 |
| CORT total dose (mg) | Insufficient data (n=4) | | | |
| Baseline platelet count (x10^9^) | 0.206 [−0.087, 0.498] | 0.143 | 10 | 46 |
| Baseline AST levels (IU/L) | −0.0054 [−0.064, 0.053] | 0.821 | 7 | 99 |
| Baseline ALT levels (IU/L) | Insufficient data (n=4) | | | |
| Baseline LDH levels (IU/L) | −0.0034 [−0.023, 0.0164] | 0.685 | 8 | 86 |
| Baseline mean blood pressure | −0.069 [−1.048, 0.909] | 0.862 | 7 | 107 |
| Baseline urinary output (ml/h) | Insufficient data (n=4) | | | |
| Blood transfusion (% required) | −0.198 [−1.769, 1.373] | 0.744 | 6 | 163 |
| % with comorbidities | 0.714 [0.269, 1.158] | **0.004** | 14 | 21 |
| Abbreviations: ALT, alanine aminotransferase; AST, aspartate aminotransferase; CI, confidence interval; CORT, corticosteroid; ICU, intensive care unit, IU, international unit; L, liter; LDH, lactic dehydrogenase; mg, milligram, ml, milliliter, n, number of included studies/datase ts; SE, standard error; UO, urinary output. | | | | |

| **S7 Table: Predictors and prognostic factors of the change in urinary output after CORT treatment in HELLP syndrome patients** | | | | |
| --- | --- | --- | --- | --- |
|  | **Coefficient [95% confidence interval]** | **P** | **Studies / datasets** | **tau^2^** |
| Number of study patients | −12.64 [−19.293, −5.998] | **0.005** | 7 | 384 |
| Mother’s age (years) | −26.36 [−63.74, 11.02] | 0.130 | 7 | 1349 |
| Gestation age (weeks) | −4.389 [−33.90, 25.122] | 0.718 | 7 | 2172 |
| Nulliparous percentage | −0.203 [−2.808, 2.403] | 0.840 | 6 | 2556 |
| CORT total dose (mg) | Insufficient data (n=3) | | | |
| Baseline platelet count (x10^9^) | −2.253 [−10.496, 5.989] | 0.514 | 7 | 2035 |
| Baseline AST levels (IU/L) | 0.683 [−0.298, 1.664] | 0.126 | 6 | 1335 |
| Baseline ALT levels (IU/L) | Insufficient data (n=3) | | | |
| Baseline LDH levels (IU/L) | 0.0798 [−0.139, 0.299] | 0.369 | 6 | 2213 |
| Baseline mean blood pressure | −0.222 [−7.362, 6.918] | 0.939 | 7 | 2233 |
| Baseline urinary output (ml/h) | −0.574 [−2.260, 1.112] | 0.422 | 7 | 1939 |
| Blood transfusion (% required) | Insufficient data (n=3) | | | |
| Intensive care stay (days) | Insufficient data (n=3) | | | |
| % with comorbidities | −0.511 [−3.899, 2.876] | 0.737 | 10 | 1527 |
| Abbreviations: ALT, alanine aminotransferase; AST, aspartate aminotransferase; CI, confidence interval; CORT, corticosteroid; ICU, intensive care unit, IU, international unit; L, liter; LDH, lactic dehydrogenase; mg, milligram, ml, milliliter, n, number of included studies/datase ts; SE, standard error; UO, urinary output. | | | | |
